# Postprandial metabolite profiles associated with type 2 diabetes clearly stratify individuals with impaired fasting glucose

**DOI:** 10.1007/s11306-017-1307-7

**Published:** 2017-12-12

**Authors:** Ruifang Li-Gao, Renée de Mutsert, Patrick C. N. Rensen, Jan Bert van Klinken, Cornelia Prehn, Jerzy Adamski, Astrid van Hylckama Vlieg, Martin den Heijer, Saskia le Cessie, Frits R. Rosendaal, Ko Willems van Dijk, Dennis O. Mook-Kanamori

**Affiliations:** 1 Department of Clinical Epidemiology, Leiden University Medical Center, P. O. Box 9600, 2300 RC Leiden, The Netherlands; 2 Division of Endocrinology, Department of Medicine, Leiden University Medical Center, Leiden, The Netherlands; 3 Einthoven Laboratory for Experimental Vascular Medicine, Leiden University Medical Center, Leiden, The Netherlands; 4 Institute of Experimental Genetics, Genome Analysis Center, Helmholtz Zentrum München, Neuherberg, Germany; 5 German Center for Diabetes Research, Neuherberg, Germany; 6 Lehrstul für Experimentelle Genetik, Technische Universität München, Freising-Weihenstephan, Germany; 7 Department of Medical Statistics and Bioinformatics, Leiden University Medical Center, Leiden, The Netherlands; 8 Department of Human Genetics, Leiden University Medical Center, Leiden, The Netherlands; 9 Department of Public Health and Primary Care, Leiden University Medical Center, Leiden, The Netherlands

**Keywords:** Metabolomics, Fasting, Postprandial, LASSO regularised logistic regression, Impaired fasting glucose, Risk stratification, Type 2 diabetes

## Abstract

**Introduction:**

Fasting metabolite profiles have been shown to distinguish type 2 diabetes (T2D) patients from normal glucose tolerance (NGT) individuals.

**Objectives:**

We investigated whether, besides fasting metabolite profiles, postprandial metabolite profiles associated with T2D can stratify individuals with impaired fasting glucose (IFG) by their similarities to T2D.

**Methods:**

Three groups of individuals (age 45–65 years) without any history of IFG or T2D were selected from the Netherlands Epidemiology of Obesity study and stratified by baseline fasting glucose concentrations (NGT (n = 176), IFG (n = 186), T2D (n = 171)). 163 metabolites were measured under fasting and postprandial states (150 min after a meal challenge). Metabolite profiles specific for a high risk of T2D were identified by LASSO regression for fasting and postprandial states. The selected profiles were utilised to stratify IFG group into high (T2D probability ≥ 0.7) and low (T2D probability ≤ 0.5) risk subgroups. The stratification performances were compared with clinically relevant metabolic traits.

**Results:**

Two metabolite profiles specific for T2D (n_fasting_ = 12 metabolites, n_postprandial_ = 4 metabolites) were identified, with all four postprandial metabolites also being identified in the fasting state. Stratified by the postprandial profile, the high-risk subgroup of IFG individuals (n = 72) showed similar glucose concentrations to the low-risk subgroup (n = 57), yet a higher BMI (difference: 3.3 kg/m^2^ (95% CI 1.7–5.0)) and postprandial insulin concentrations (21.5 mU/L (95% CI 1.8–41.2)).

**Conclusion:**

Postprandial metabolites identified T2D patients as good as fasting metabolites and exhibited enhanced signals for IFG stratification, which offers a proof of concept that metabolomics research should not focus on the fasting state alone.

**Electronic supplementary material:**

The online version of this article (10.1007/s11306-017-1307-7) contains supplementary material, which is available to authorized users.

## Introduction

With rapid advances in high throughput mass spectrometry-based techniques, metabolomics is emerging as an important approach in clinical research for obesity and T2D (Wang et al. 2011; Floegel et al. 2013). To date, most metabolomics research is performed under fasting conditions. Nonetheless, studies have indicated that postprandial metabolic disturbances might be novel risk factors for disease development (Mathew et al. 2014). Therefore, using both fasting and postprandial metabolite measurements will extend the knowledge on flexibility of the human metabolome and on metabolic pathways involved in the initiation and progression of T2D.

Impaired fasting glycaemia (IFG) and/or impaired glucose tolerance (IGT) is considered a pre-diabetic state. Several major clinical trials showed a reduction of T2D risk in the populations with IFG and/or IGT by lifestyle or pharmacologic interventions (Nathan et al. 2007). However, not all participants benefited from interventions and not all individuals who did not receive an intervention progressed to T2D, underlining the heterogeneity of the IFG/IGT group (Dunkley et al. 2014). These findings warrant the search for cost-effective risk stratification approaches to decide who to treat and under what circumstances. Although an abnormal oral-glucose tolerance test (OGTT) result is predictive for T2D, this test only assesses one component of metabolism, namely glucose metabolism. Previous longitudinal studies designed and validated a model comprised of six blood biomarkers to assess the 5-year risk of developing T2D (Kolberg et al. 2009; Urdea et al. 2009). However, very few studies had explored the potential of IFG stratification with postprandial metabolites in a drug-naïve population.

In this study, we undertook systematic analyses of 163 blood circulating metabolites under fasting, postprandial states and also the responses (postprandial–fasting) between them. In different states, we firstly aimed to identify a subset of metabolites with the best classification performance to distinguish untreated T2D from NGT individuals. Then, we utilized the selected metabolite profile under fasting state to stratify IFG individuals according to their T2D similarities as an empirical benchmark. Furthermore, the stratification performances of postprandial and response metabolite profiles were compared to the stratification performance of fasting metabolite profile.

## Materials and methods

### Study design

The study was embedded in a population-based prospective cohort, the Netherlands Epidemiology of Obesity (NEO) study (de Mutsert et al. 2013). All participants gave written informed consent and the Medical Ethical Committee of the Leiden University Medical Center (LUMC) approved the study design. Initiated from 2008, men and women aged between 45 and 65 years with a self-reported body mass index (BMI) of 27 kg/m^2^ or higher living in the greater area of Leiden (in the west of the Netherlands) were eligible to participate in the NEO study. Participants were invited for a baseline visit at the NEO study center in the LUMC after an overnight fast. At the baseline visit, fasting blood samples were drawn. Within the next 5 min after the fasting blood draw, a liquid mixed meal (400 mL, 600 kcal, with 16% of energy (En%) derived from protein, 50 En% carbohydrates, and 34 En% fat) was consumed and subsequent blood samples were drawn 30 and 150 min after the meal.

### Population and diabetes classification

**Fig. 1:**
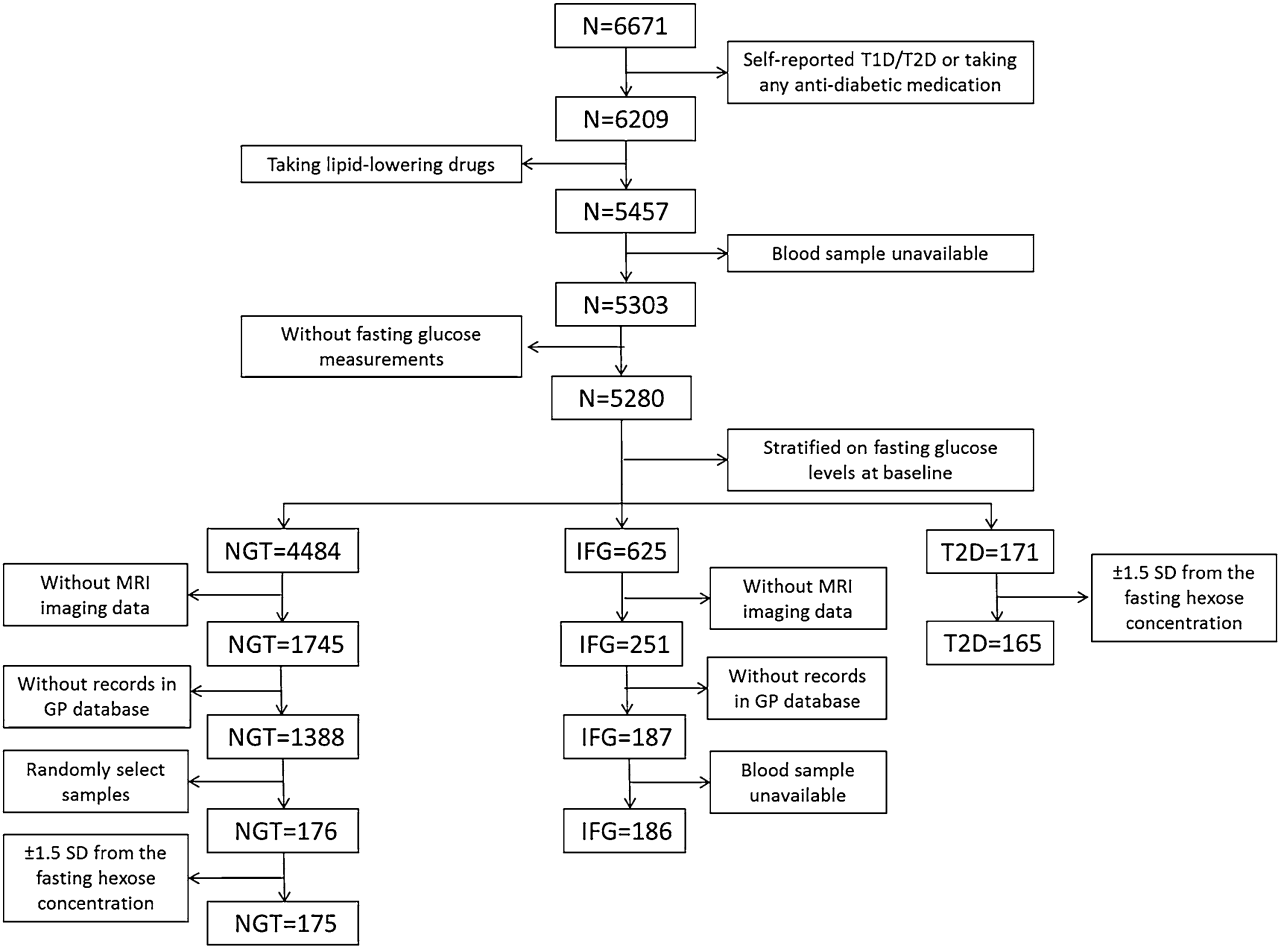
Flowchart of participant selection from NEO study for the current metabolomics study

From the 6671 participants included in the NEO study, individuals were selected (1) without a history of T2D or IFG and (2) without the use of any glucose- or lipid-lowering drugs (Fig. 1). Information on diabetes status at baseline was verified via medical records of the general practitioners of the participants. A history of diabetes was defined as the presence of a diagnosis coded in the medical records with International Classification of Primary Care (ICPC) codes T90 (diabetes mellitus, any type), T90.1 (type 1 diabetes mellitus) or T90.2 (type 2 diabetes mellitus) before the baseline study visit. A history of IFG was defined according to the presence of ICPC codes A91.05 or B85.01 (both impaired glucose tolerance) in absence of codes T90, T90.1 or T90.2, before the baseline study visit.

The participants were further classified into three groups as described by the World Health Organization (Definition and diagnosis of diabetes mellitus and intermediate hyperglycemia 2006). Newly diagnosed T2D was defined as having a fasting glucose concentration ≥ 7.0 mmol/L at the baseline measurement without a previous history of T2D. Newly diagnosed IFG was defined as having a fasting glucose concentration ≥ 6.1 and < 7.0 mmol/L at the baseline measurement without a previous history of having IFG or T2D. Participants with a fasting glucose concentration ≤ 6.0 mmol/L were defined as having a normal glucose tolerance (NGT).

As an additional quality control step, we compared fasting glucose concentrations with fasting hexose concentrations (which contains > 90% glucose). Samples were excluded if the fasting glucose concentration deviated more than ± 1.5 standard deviation from the fasting hexose concentration (n = 7).

### Metabolomics

Metabolomic measurements were performed in both fasting and postprandial (t = 150 min after the meal) EDTA-plasma samples at the Genome Analysis Center at the Helmholtz Zentrum München, Germany, using the Biocrates Absolute*IDQ*™ p150 assay and FIA-ESI-MS/MS (flow injection-electrospray-triple quadrupol mass spectrometry) measurements. Due to the budget constraints and the fact that metabolite levels at 150 min change more significantly than 30 min after the meal (Krug et al. 2012), there were no metabolomic measurements at 30 min. The p150 assay includes 163 metabolites (Supplementary Table S1) from five substance classes: acylcarnitines (n = 41), sphingolipids (n = 15), glycerophosphocholines (n = 92), amino acids (n = 14; 13 proteinogenic amino acids and ornithine) and hexoses (sum of glucose, galactose and fructose). The method of Biocrates Absolute*IDQ*™ p150 assay has been proven to be in conformance with the EMEA-Guideline (Guideline on bioanalytical method validation. EMEA/CHMP/EWP/192217/2009 Rev. 1 Corr. 2 2011), which implies a proof of reproducibility within a given error range. The assay as well as the metabolite denomination have been described in details before (Romisch-Margl et al. 2012). Mass spectrometric analyses were done on an API 4000 triple quadrupole system (Sciex Deutschland GmbH, Darmstadt, Germany) equipped with a 1200 Series HPLC (Agilent Technologies Deutschland GmbH, Böblingen, Germany). Metabolite concentrations were calculated using internal standards and reported in µM. Three metabolites (PC aa C30:2, PC ae C38:1, SM C22:3) were dropped out from the analyses due to under the detection limit. Hexose (H1) was also not considered in the metabolite profile selection as a result of its high correlation to the fasting glucose concentration, leaving 159 metabolites for the analyses. The measurement of other blood parameters (glucose, insulin, HbA1c, and the lipid profile) has been described previously (de Mutsert et al. 2013).

### Metabolite profile selection

Our preliminary step was to identify fasting, postprandial and response metabolite profiles, which could distinguish between the newly diagnosed T2D patients and NGT individuals. We used the least absolute shrinkage and selection operator (LASSO) method for metabolite profile selection (Supplementary information). Compared to standard logistic regression, LASSO method adds a constraint, i.e. it demands that the sum of all the parameters is smaller than a value λ. The selection of λ is performed through ten-fold cross validation (CV) by optimizing the classification performance measured by area under the curve (AUC). Due to the restriction to value λ, some of the parameters will be shrunken to zero, which means the AUC is not improved by taking the corresponding metabolite into account. Put together, the variables with non-zero estimated parameters form a metabolite profile.

In the fasting and postprandial states, metabolite concentrations were log-transformed to obtain normal distributions. Response was defined as log-transformed difference of metabolite concentrations between postprandial and fasting state (i.e. log[Metabolite_t=150_] − log[Metabolite_t=0_]). All transformed values were Z-score normalized (with a mean of zero and a standard deviation of one), in order to keep the same variance across different metabolites in the LASSO model. Additionally, all the selected metabolites were checked individually for their concentration differences between the NGT and T2D group on their original untransformed scales (right skewed distributions) by two-sided Wilcoxon rank-sum tests with Bonferroni correction (p values < 0.05/the number of metabolites selected at least once in a metabolite profile) for multiple testing.

### Stratification of IFG individuals

The previously selected fasting, postprandial and response metabolite profiles were utilised separately to stratify the IFG individuals into subgroups by their metabolite profile similarities to T2D. Assuming the stratification performance by fasting metabolite profile as an empirical benchmark, the consistency of predicted probabilities among different prandial states were checked by Pearson correlation. Taking the left-skewed probability distributions into account, the IFG group was further trichotomized with the rules: (1) if the predicted probabilities to be T2D were ≥ 0.7 (above the average probability prediction), the individuals were classified as high-risk of T2D and annotated as the predicted disease (PD); (2) if the predicted probabilities were ≤ 0.5 (below the average probability prediction), the individuals were classified as low-risk of T2D and labelled as the predicted normal (PN); (3) for all the remaining individuals, they were classified as predicted intermediate risk (PM). Subsequently, BMI, HbA1c, fasting/postprandial glucose and insulin concentrations, as well as HOMA-IR and HOMA-β were compared across the three predicted groups (predicted low, intermediate and high risk). The differences between the predicted three groups were tested by one-way ANOVA with Tukey post-hoc using PN group as the reference.

All statistical analyses were performed in R version 3.0.3. LASSO models were derived by R glmnet package (Friedman et al. 2010). Wilcoxon rank-sum tests were performed by wilcox.test function and ANOVA was tested by aov and TukeyHSD function in R stats package.

## Results

Table 1 summarizes the baseline characteristics of all the participants, stratified by their fasting glucose levels into three groups (NGT (n = 176), IFG (n = 186), T2D (n = 171)). The average age was slightly younger in the NGT group than in the newly diagnosed IFG and T2D group. The average BMI was lower in the NGT group (28.3 kg/m^2^) than in the IFG and T2D group (30.6 and 32.6 kg/m^2^, respectively).

**Table 1:** Baseline characteristics of the study population, stratified by fasting glucose levels

	NGT (n = 175)	IFG (n = 186)	T2D (n = 165)	p values^a^
Demographic/anthropometric				
Age (years)	55.1 (5.6)	56.8 (5.8)	56.4 (5.5)	< 0.05
Sex (% men)	84 (48.0)	118 (63.4)	90 (54.5)	< 0.05
BMI (kg/m^2^)	28.3 (4.8)	30.6 (4.2)	32.6 (5.2)	< 0.05
Fasting blood concentrations				
Glucose (mmol/L)	5.2 (0.5)	6.4 (0.2)	8.1 (1.8)	< 0.05
Insulin (mU/L)	11.3 (8.2)	15.4 (8.3)	22.0 (24.0)	< 0.05
HbA1c (%)	5.3 (0.2)	5.5 (0.3)	6.3 (1.1)	< 0.05
HDL-cholesterol (mmol/L)	1.5 (0.4)	1.4 (0.4)	1.2 (0.3)	< 0.05
Total cholesterol (mmol/L)	5.9 (1.1)	6.0 (1.0)	5.9 (1.1)	0.8
Triglycerides (mmol/L)	1.2 (0.8)	1.8 (1.3)	1.9 (1.0)	< 0.05
LDL-cholesterol (mmol/L)	3.8 (1.0)	3.8 (0.9)	3.8 (1.0)	0.9
Insulin resistance (HOMA-IR)	2.7 (2.0)	4.4 (2.4)	7.8 (8.6)	< 0.05
Beta-cell function (HOMA-β)	130.9 (90.1)	106.7 (56.4)	103.8 (111.1)	< 0.05

Values represent mean (standard deviation), or n (%)

Normal glucose tolerance (NGT): fasting glucose ≤ 6.0 mmol/L

Impaired fasting glycaemia (IFG): fasting glucose ≥ 6.1 and < 7.0 mmol/L

Type 2 diabetes (T2D): fasting glucose ≥ 7.0 mmol/L

^a^For continuous variables, p values were derived from the one-way ANOVA test; for categorical variable, p values were derived from chi-squared test. For glucose, insulin, triglycerides, HOMA-IR and HOMA-β, the raw values were log-transformed before the test, in order to obtain a normal distribution

**Fig. 2:**
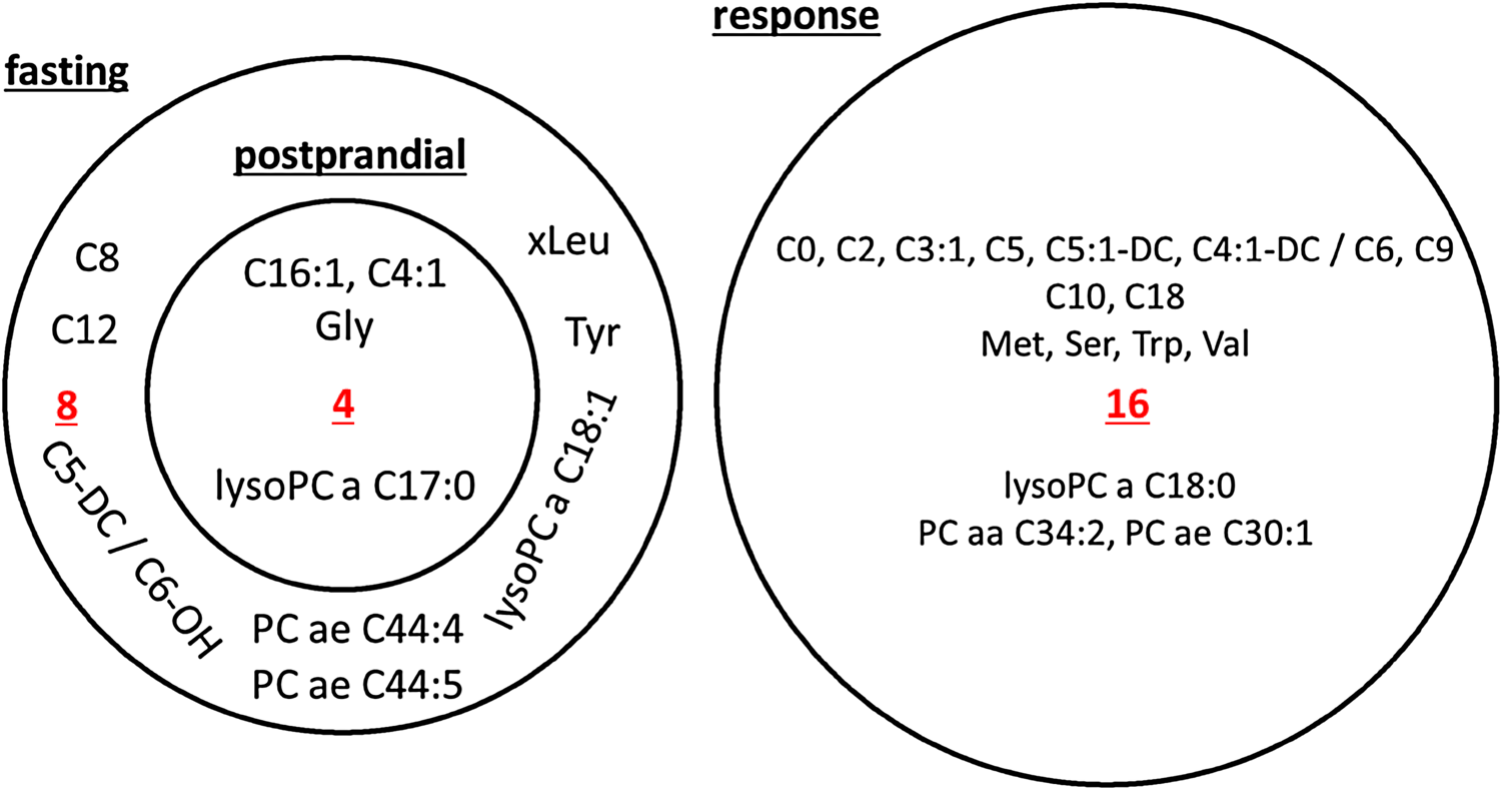
The metabolite profiles selected by LASSO regularised logistic regression and the Venn diagram of the most parsimonious metabolite profiles under fasting, postprandial and response. The numbers highlighted in red colour indicate the number of metabolites composed of the profile. Full metabolite names are shown in Supplementary Table S1

Under the fasting and postprandial state, the most parsimonious profile was composed of 12 and 4 metabolites, respectively. The 4 metabolites selected under postprandial state fully overlapped with the metabolite profile under fasting state (Fig. 2). The most parsimonious profile selected under the response comprised of 16 metabolites that were unique. Acylcarnitines, mainly the short-chain acylcarnitines accounted for over half (9 out of 16) of the metabolites in the response profile.

Using Wilcoxon rank-sum test and adjusting for multiple testing, individual metabolite concentrations were compared between the NGT and T2D groups. Table 2 summarizes the results of comparing metabolite concentrations between the NGT and T2D groups for the 28 metabolites selected at least once in a metabolite profile. In the fasting state, 9 out of 12 metabolites showed significant differences between the two groups. Amongst them, the median concentration of C16:1 was 26.53% (95% CI [22.33, 30.83%]) higher in the T2D group as compared with the NGT group. In contrast, the median concentration of lysoPC a C17:0 was 21.06% (95% CI [− 15.40, − 26.29%]) lower in the T2D group than the NGT group. Under the postprandial state, all the four selected metabolites were found to discriminate the T2D from the NGT group, with the largest difference being for C16:1, which was 29.88% (95% CI [25.28, 35.07%]) higher in the T2D group than the NGT group. In the response profile, however, unlike the fasting and postprandial states, only five amino acids (namely Gly, Met, Ser, Val and xLeu) together with C10, out of sixteen selected metabolites, were significantly different in the T2D group compared to the NGT group.

**Table 2:** Results of the Wilcoxon rank-sum tests of metabolite concentration difference between the NGT and T2D groups

Metabolite	Fasting			Postprandial			Response		
	Hit	%diff	p value	hit	%diff	p value	Hit	%diff	p value
C0		7.46	1.85E−03		4.19	5.25E−02	x	− 2.43	2.94E−02
C10		6.54	7.17E−02		17.51	**1.13E**−**06** ^a^	x	10.28	**1.51E**−**03** ^a^
C12	×	5.49	1.24E−01		11.10	**9.54E**−**04** ^a^		4.54	1.06E−01
C16:1	×	26.53	**1.74E**−**28** ^a^	×	29.88	**2.75E**−**35** ^a^		3.45	1.09E−02
C18		5.61	6.76E−02		2.59	3.77E−01	×	− 3.39	6.66E−02
C2		6.85	3.70E−02		12.50	**8.38E**−**05** ^a^	×	5.71	2.94E−02
C3:1		− 4.32	6.82E−02		3.63	1.53E−01	×	6.86	2.76E−02
C4:1	×	16.40	**6.01E**−**16** ^a^	×	20.02	**7.52E**−**16** ^a^		3.05	2.06E−01
C5		13.47	**4.32E**−**05** ^a^		18.23	**2.49E**−**08** ^a^	×	4.68	3.60E−02
C5:1-DC		4.52	5.82E−02		10.73	**8.93E**−**06** ^a^	×	6.30	4.20E−02
C5-DC/C6-OH	×	− 5.13	2.34E−02		− 5.16	2.55E−02		1.61	5.66E−01
C4:1-DC/C6		9.83	**8.15E**−**04** ^a^		7.95	**1.49E**−**03** ^a^	×	− 2.66	2.35E−01
C8	×	2.47	4.18E−01		4.91	8.57E−02		1.96	4.49E−01
C9		1.58	6.54E−01		8.29	1.17E−02	×	6.49	4.06E−03
Gly	×	− 13.37	**1.20E**−**08** ^a^	×	− 18.37	**2.25E**−**14** ^a^		− 4.80	**2.11E**−**04** ^a^
Met		2.87	8.55E−02		− 6.77	**1.03E**−**03** ^a^	×	− 10.25	**1.64E**−**07** ^a^
Ser		− 6.51	**7.40E**−**04** ^a^		− 14.97	**8.02E**−**12** ^a^	×	− 9.72	**6.39E**−**11** ^a^
Trp		3.86	**1.29E**−**03** ^a^		3.91	**5.96E**−**04** ^a^	×	0.20	8.53E−01
Tyr	×	14.94	**1.93E**−**11** ^a^		9.29	**5.56E**−**05** ^a^		− 5.02	7.14E−03
Val		− 1.69	3.62E−01		− 6.51	**6.68E**−**04** ^a^	×	− 6.60	**3.56E**−**05** ^a^
xLeu	×	16.68	**2.52E**−**11** ^a^		9.04	**2.12E**−**04** ^a^		− 6.58	**1.47E**−**03** ^a^
lysoPC a C17:0	×	− 21.06	**7.50E**−**13** ^a^	×	− 21.72	**7.59E**−**14** ^a^		− 0.64	6.71E−01
lysoPC a C18:0		− 7.63	3.10E−03		− 9.42	**1.04E**−**04** ^a^	×	− 2.03	1.04E−01
lysoPC a C18:1	×	− 14.66	**1.59E**−**07** ^a^		− 15.35	**2.98E**−**09** ^a^		− 2.09	1.97E−01
PC aa C34:2		7.10	**2.37E**−**05** ^a^		8.58	**1.51E**−**07** ^a^	×	1.78	8.45E−02
PC ae C30:1		6.81	1.21E−01		0.87	8.38E−01	×	− 5.68	2.60E−01
PC ae C44:4	×	− 8.53	**4.60E**−**04** ^a^		− 8.60	**2.45E**−**04** ^a^		− 0.37	7.52E−01
PC ae C44:5	×	− 9.61	**1.94E**−**04** ^a^		− 11.05	**2.79E**−**05** ^a^		− 1.55	1.12E−01

The original untransformed metabolite concentrations are used to test the statistical significance between NGT and T2D for individual metabolite

The composition of each metabolite profile selected by LASSO model is specified by “×” in the hit column

%diff reflects the percentage of metabolite concentration difference between the NGT and T2D group. It is calculated by taking the median of metabolite concentration differences between the NGT and T2D group divided by the median metabolite concentration in NGT group. The ratio multiplies by 100 to percentage

p values are calculated by two-sided Wilcoxon rank-sum tests

^a^p values < 1.79 × 10^−3^ (= 0.05/28 for Bonferroni correction) for significant difference between NGT and T2D group, and highlighted in bold

**Fig. 3:**
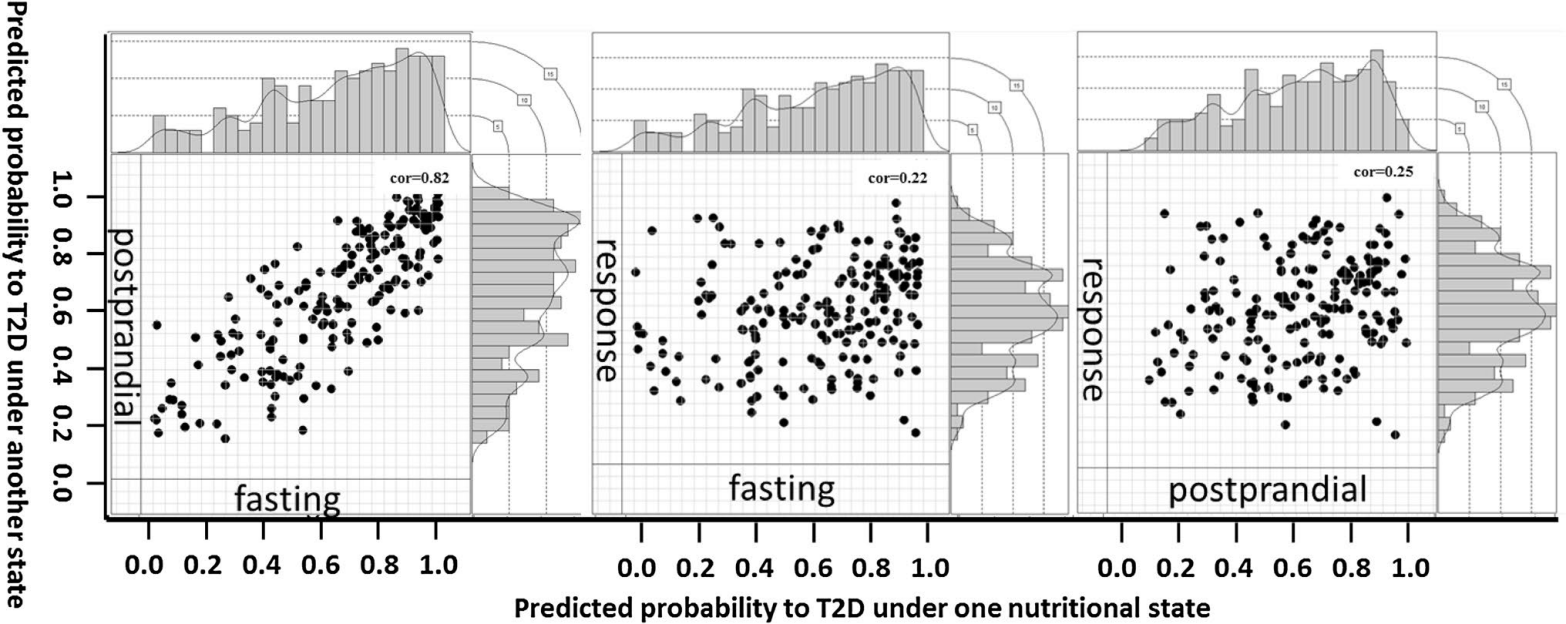
The distributions of T2D probability prediction for the IFG group under fasting, postprandial and response and the probability correlations between different states. The Pearson correlations were labeled on the top of the figure

**Fig. 4:**
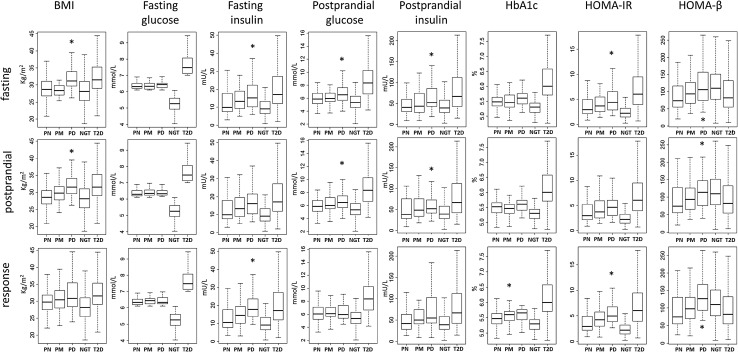
The distributions of clinically relevant metabolic traits across three subgroups (*PN* predicted normal, *PM* predicted middle, *PD* predicted T2D) in the IFG group predicted by LASSO model, along with the NGT and T2D group. Differences between PN, PM and PD were statistically depicted by one-way ANOVA with Tukey post hoc from PN as the reference group. *p value < 0.05

Using the selected fasting, postprandial and response metabolite profiles separately, 186 IFG individuals were stratified into three subgroups (predicted low, intermediate, and high risk to T2D) based on their metabolite profile similarities to T2D. Under fasting and postprandial states, the predicted probabilities were highly consistent (correlation coefficient 0.82; 95% CI [0.76, 0.86]), and 130 out of 186 IFG individuals were predicted to be in the same risk category by two different states. Only four cases were assigned inconsistently from either high to low risk category or from low to high risk category. However, the predicted probabilities by response profile showed a large discrepancy (Fig. 3). To verify the stratification performance by different profiles, we assessed clinically relevant metabolic traits within the three IFG subgroups (Fig. 4). Stratified by the postprandial profile composed of four metabolites alone, the predicted high-risk group (PD) revealed significant difference in BMI (difference = + 3.32 kg/m^2^, 95% CI [1.67, 4.97]), postprandial glucose (difference = + 0.74 mmol/L, 95% CI [0.17, 1.31]) and insulin (difference = + 21.51 mU/L, 95% CI [1.80, 41.22]) concentrations, as well as HOMA-β (difference = + 23.83, 95% CI [0.44, 47.21]) compared with the low-risk group (PN). By the response profile (with the metabolite profile composed of sixteen metabolites), the predicted high-risk group displayed a higher fasting insulin (7.21 mU/L, 95% CI [3.08, 11.33]), HOMA-IR (2.13, 95% CI [0.93, 3.33]) and HOMA-β (46.95, 95% CI [18.73, 75.17]) than PN group. Interestingly, for all three stratifications (by fasting, postprandial and response metabolite profiles separately), fasting glucose concentration was not shown higher levels in the predicted disease group than in the predicted normal group.

## Discussion

Nearly all the endeavours on metabolomics research in T2D have focused on fasting metabolites alone. In this study, we found that by only four metabolites in the postprandial state, instead of twelve in the fasting state, could stratify IFG individuals with similar efficiency. Furthermore, the predicted T2D probabilities for the IFG individuals based on the selected fasting/postprandial metabolite profile were positively associated with other metabolic traits, such as higher body mass index and insulin concentration.

### Discriminative metabolite profile in the fasting state

There is growing evidence that increased levels of plasma acylcarnitines are associated with the risk of T2D (Mihalik et al. 2010). In the current study, C16:1 and C4:1, as well as some other intermediate fatty acid beta-oxidation by-products (e.g. C8, C12) were identified under the fasting state and observed to be with higher concentrations in the T2D group compared to the NGT group. Likewise, the glucogenic amino acids glycine and serine can be converted into glucose by gluconeogenesis, predominantly in the fasting state. In our study, the concentration of glycine was significantly decreased among the T2D individuals compared to the NGT group and selected by the LASSO model. This finding further supports the hypothesis that with the development of T2D, insulin exerts a decreased inhibitory effect on hepatic gluconeogenesis and as a result leads to an increased demand and consumption of glucogenic amino acids (Renner et al. 2012). Additionally, consistent with the previous findings, plasma lysophosphatidylcholines (e.g. lysoPC a 18:0, lysoPC a 18:1 and lysoPC a 18:2) (Barber et al. 2012) were inversely associated with the T2D risk. However, the selection of the lysophosphatidylcholines (lysoPC a C17:0 and lysoPC a C18:1) in the present metabolite profile might be due to the difference in adiposity rather than diabetes status per se (Barber et al. 2012) as BMI was also observed to be higher in the T2D group than the NGT group in the current study population. Although common confounders, such as BMI in the current study, are generally not taken into consideration in the prediction research (van Diepen et al. 2017), oversampling on the overweighed and obese population in the current study may hinder the generalizability of the findings. However, after regressing out age, sex and BMI from the metabolite concentrations, we still observed very similar results regarding IFG predictions under different states (data not shown).

### Discriminating metabolites in the postprandial and response states

For the postprandial state, a profile consisting of only four metabolites could distinguish the TD2 group from the NGT group. Thus, a controlled meal challenge greatly enhanced the metabolite signals to separate the T2D from the NGT individuals. The fact that the four metabolites are able to differentiate in a non-fasting state provides proof of concept for the potential clinical usefulness of non-fasting metabolites as biomarkers.

It is important to note that > 50% of the T2D-specific metabolites from the response were acylcarnitines, and more specifically short-chain acylcarnitines. Acetylcarnitine (C2), the shortest acylcarnitine, has been identified as one of the fasting state biomarkers for diagnosis of pre-diabetes (Wang-Sattler et al. 2012). It was described to be involved in substrate selection and promotion of metabolic flexibility (Schooneman et al. 2013). However, we did not observe a significant difference in the fasting C2 concentration between the NGT and T2D group from the present dataset. In contrast, a significant C2 concentration increase was found in the postprandial state.

### Stratification IFG individuals by metabolite profile similarity to T2D

By taking stratification performance of fasting metabolite profile as an empirical benchmark, 130 out of 186 IFG individuals were assigned to the same risk categories by the postprandial metabolite profile. Of the remaining 56 “misclassified” individuals, only three cases were predicted to be low-risk by applying the postprandial metabolite profile, which were assigned as high risk by the fasting metabolite profile. In a clinical setting, only the predicted high-risk individuals would be taken into account for an intervention program. So compared to the fasting metabolite profile, the postprandial metabolite profile achieved a very similar risk stratification. The categories for NGT, IFG and T2D were defined by the fasting glucose measurements at baseline. However, in the subgroups of the IFG individuals stratified by the selected metabolite profiles, we were unable to observe a clear distinction based on the fasting glucose levels. Nevertheless, there was a clear distinction in the fasting and postprandial insulin concentrations across three subgroups in the IFG stages, stratified by different profiles. So insulin levels might be more sensitive than the fasting glucose levels to evaluate the progression of disease with respect to glucose and lipid metabolism, especially in the pre-diabetic stage. For the other clinically relevant glycaemic traits, such as HbA1c, HOMA-IR, HOMA-β, the high-risk subgroup also displayed increased levels compared to the low-risk subgroup, which further confirmed the effectiveness of metabolite profiles in the IFG stratification.

### Methodological considerations

Some methodological issues should be considered. Firstly, a major strength of the current study is that all participants were naïve to drug treatment. In most cross-sectional metabolomics studies on T2D, the participants were using glucose- or lipid-lowering drugs, which could influence the results (Altmaier et al. 2014). Secondly, many metabolomics studies on T2D confirm the diagnosis based on questionnaire or by a physician. We defined newly diagnosed T2D by the fasting glucose levels. Unfortunately, we only had a single measurement and no oral glucose tolerance test as is recommended by the World Health Organization. Therefore, there may be some misclassification in the diabetes classification. The main limitation is related to our cross-sectional study design. In this analysis, we could not affirm whether the predicted high-risk subgroup in IFG had a larger proportion of individuals developing to T2D than the low-risk subgroup. Besides, due to our definition, we are unable to investigate patients with IGT. Subsequently, we cannot extrapolate our results to the entire pre-diabetic population at risk of developing T2D.

## Conclusion

In conclusion, postprandial metabolite profiles revealed enhanced signals to distinguish the NGT and T2D individuals and provided a very similar IFG stratification scheme as the fasting metabolite profile, which offers a proof of concept that metabolomics research on type 2 diabetes should not be focused on the fasting state alone. Follow-up studies, such as in the NEO study, will allow us to investigate whether predicted high-risk IFG individuals truly develop to the disease.

## Electronic supplementary material

Below is the link to the electronic supplementary material.

Supplementary material 1 (DOCX 287 KB)
