# Supplementary material for: Postprandial metabolite profiles associated with type 2 diabetes clearly stratify individuals with impaired fasting glucose
Source: Metabolomics. 2017 Dec 12;14(1):13. doi: 10.1007/s11306-017-1307-7 (PMC5727148; doi:10.1007/s11306-017-1307-7)
Supplement: Supplementary file 1 — Supplementary material 1 (DOCX 287 KB) [file 11306_2017_1307_MOESM1_ESM.docx]

**Supplementary information**

**Selecting the tuning parameter λ in LASSO models**

The selection of parameter λ is based on the evaluation of classification performance (reflected by the area under the curve (AUC) in the current study) at each value of λ. Normally a ten-fold cross-validation is used, where 90% of all the samples are randomly selected to form a training set for parameter estimation and the other 10% of the samples are kept for model validation. Since the folds are selected at random, the determination of λ will be different from time to time, which further leads to diverse metabolite profile compositions accordingly. One of the solutions to reduce the randomness is to repeat the cross-validation for multiple times. In our analysis, we repeated the ten-fold cross-validation for ten times, and chose the metabolite profile with the smallest number of metabolites (Fig. S1). The reasons for choosing the most parsimonious set of metabolites lie in two aspects: 1). The metabolites comprised of the most parsimonious set were also selected nearly 10/10 times, which were deemed as the most robust makers; 2). The redundant or irrelevant metabolites might be no harm for a model from the classification point of view, but they are not economical for practical use.

Commonly a “one-standard error” rule is adopted to determine the optimal parameters within cross-validation, in which the most parsimonious model is chosen with the AUC no more than one standard error above the AUC of the “best” model (AUC achieved the maximum by the parameter) (Hastie et al. 2009). By this rule, the most regularized model is determined, which reduces the chance of overfitting. Fig. S2 shows the AUCs achieved along with varying cut-off values on λ as well as the corresponding number of metabolites selected in the most parsimonious models highlighted in Fig. S1. On the basis of the “one-standard error” rule, the determination of λ was right shift (towards larger λ values and less number of metabolites) from the maximum of AUCs. Under the fasting state, twelve metabolites were obtained with the median AUC of 0.93 (range: [0.91, 0.94]) within ten-fold cross-validation. A similar pattern was observed for the four metabolites identified under the postprandial state, with a median AUC of 0.93 (range: [0.91, 0.94]). The sixteen metabolites selected under the response achieved a markedly lower separation between the NGT and T2D groups, with a median AUC of 0.81 (range: [0.77, 0.84]).

**References**

Hastie, T., Tibshirani, R., & Friedman, J. (2009). *The Elements of Statistical Learning: Data Mining, Inference, and Prediction.* : Springer-Verlag.


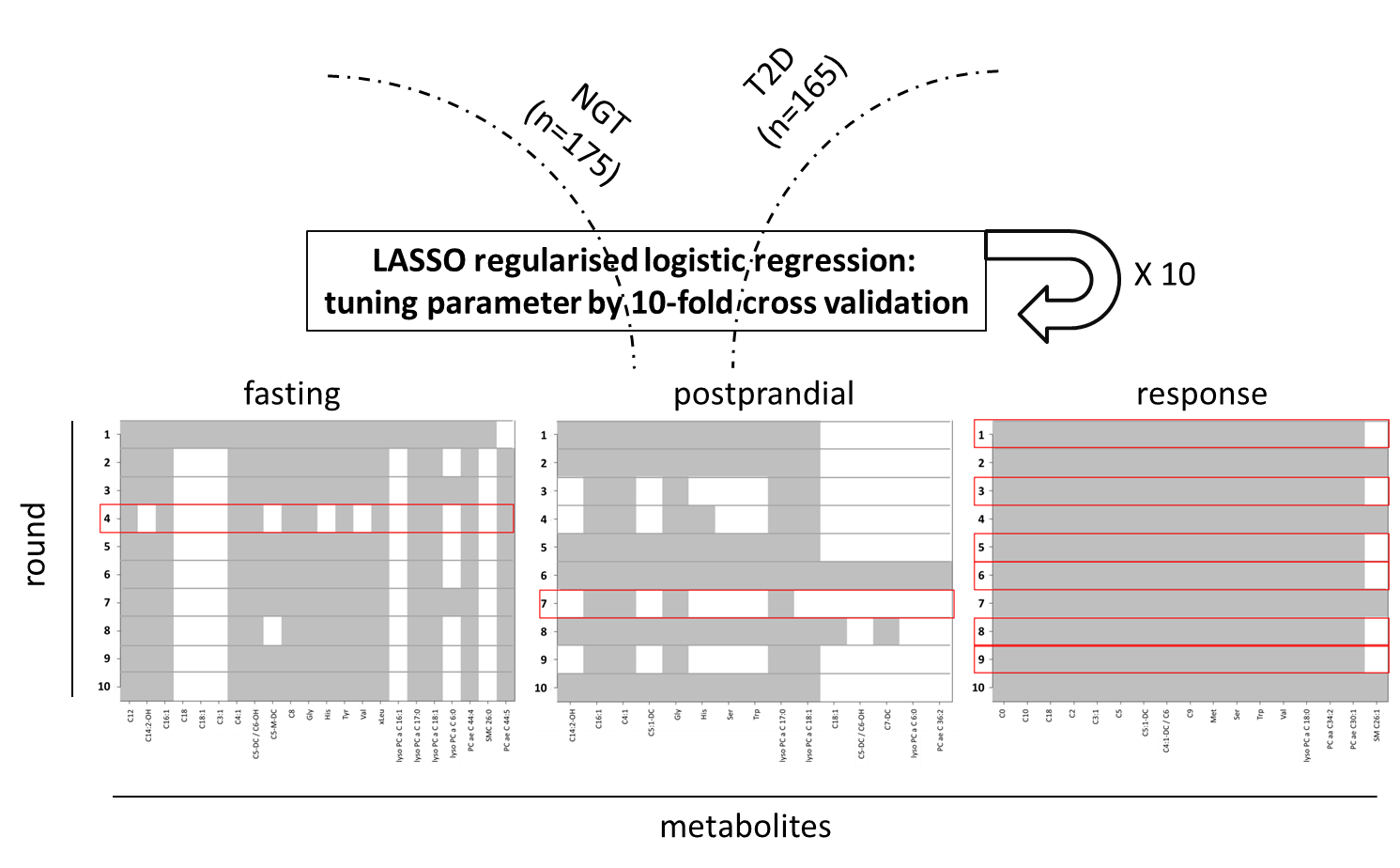


**Fig. S1** The procedure of performing LASSO regularised logistic regression. All the samples from the NGT and T2D group were used as the input for the LASSO model selection. Ten-fold cross validation was repeated by ten times, and the most parsimonious metabolite profile was highlighted in red square.


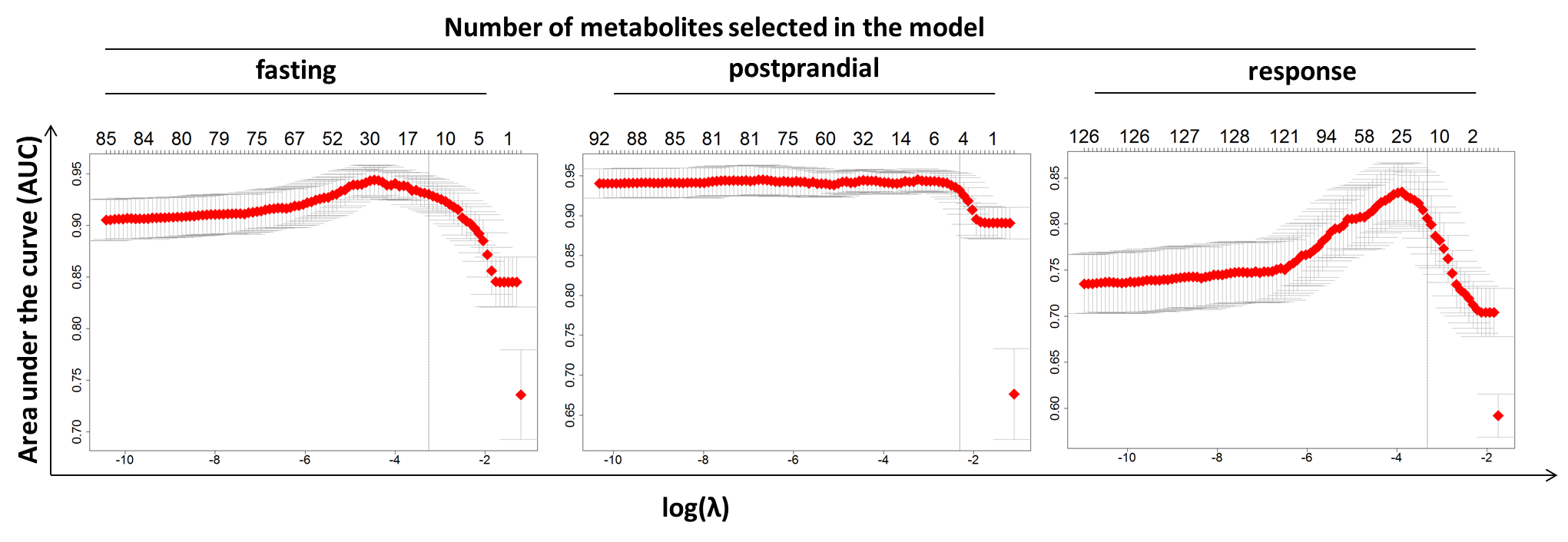


**Fig. S2** Parameter λ and the optimal number of metabolites were determined by optimizing area under the curve (AUC). The number of metabolites selected for the most parsimonious metabolite profile was indicated by the dashed line (within one standard error of the AUC maximum). The red dot represented the average value of AUC within the ten-fold cross validation, and the largest and smallest AUCs by a certain number of metabolites were labelled by the grey bar across the red dot.

**Table S1** List of metabolites measured in Biocrates platform, with the short names and biochemical names.

| **Short_name** | **Biochemical_name** | **HMDB_ID** | **KEGG_ID** |
| --- | --- | --- | --- |
| **Acylcarnitines** | | | |
| C0 | DL-Carnitine | HMDB00062 | C15025 |
| C10 | Decanoyl-L-carnitine | HMDB00651 |  |
| C10:1 | Decenoyl-L-carnitine | HMDB13205 |  |
| C10:2 | Decadienyl-L-carnitine |  |  |
| C12 | Dodecanoyl-L-carnitine | HMDB02250 |  |
| C12:1 | Dodecenoyl-L-carnitine |  |  |
| C12-DC | Dodecanedioyl-L-carnitine | HMDB13327 |  |
| C14 | Tetradecanoyl-L-carnitine | HMDB05066 |  |
| C14:1 | Tetradecenoyl-L-carnitine | HMDB02014 |  |
| C14:1-OH | Hydroxytetradecenoyl-L-carnitine | HMDB13330 |  |
| C14:2 | Tetradecadienyl-L-carnitine | HMDB13331 |  |
| C14:2-OH | Hydroxytetradecadienyl-L-carnitine |  |  |
| C16 | Hexadecanoyl-L-carnitine | HMDB00222 | C02990 |
| C16:1 | Hexadecenoyl-L-carnitine | HMDB06317 |  |
| C16:1-OH | Hydroxyhexadecenoyl-L-carnitine | HMDB13333 |  |
| C16:2 | Hexadecadienyl-L-carnitine | HMDB13334 |  |
| C16:2-OH | Hydroxyhexadecadienyl-L-carnitine | HMDB13335 |  |
| C16-OH | Hydroxyhexadecanoyl-L-carnitine |  |  |
| C18 | Octadecanoyl-L-carnitine | HMDB00848 |  |
| C18:1 | Octadecenoyl-L-carnitine | HMDB05065  HMDB06464 |  |
| C18:1-OH | Hydroxyoctadecenoyl-L-carnitine | HMDB13339 |  |
| C18:2 | Octadecadienyl-L-carnitine | HMDB06461 |  |
| C2 | Acetyl-L-carnitine | HMDB00201 | C02571 |
| C3 | Propionyl-L-carnitine | HMDB00824 | C03017 |
| C3:1 | Propenyl-L-carnitine | HMDB13124 |  |
| C3-DC / C4-OH | Malonyl-L-carnitine/Hydroxybutyryl-L-carnitine | HMDB02095  HMDB13127 |  |
| C3-DC-M / C5-OH | Methylmalonyl-L-carnitine/Hydroxyvaleryl-L-carnitine | HMDB13132 |  |
| C3-OH | Hydroxypropionyl-L-carnitine | HMDB13125 |  |
| C4 | Butyryl-L-carnitine | HMDB00736  HMDB02013 | C02862 |
| C4:1 | Butenyl-L-carnitine | HMDB13126 |  |
| C4:1-DC / C6 | Fumaryl-L-carnitine/Hexanoyl-L-carnitine | HMDB00705  HMDB00756 |  |
| C5 | Valeryl-L-carnitine | HMDB00688  HMDB13128  HMDB41993  HMDB00378 |  |
| C5:1 | Tiglyl-L-carnitine | HMDB02366 |  |
| C5:1-DC | Glutaconyl-L-carnitine | HMDB13129 |  |
| C5-DC / C6-OH | Glutaryl-L-carnitine/Hydroxyhexanoyl-L-carnitine | HMDB13130 |  |
| C5-M-DC | Methylglutaryl-L-carnitine | HMDB00552 |  |
| C6:1 | Hexenoyl-L-carnitine | HMDB13161 |  |
| C7-DC | Pimelyl-L-carnitine | HMDB13328 |  |
| C8 | Octanoyl-L-carnitine | HMDB00791 | C02838 |
| C8:1 | Octenoyl-L-carnitine | HMDB00791 | C02838 |
| C9 | Nonayl-L-carnitine | HMDB06320 |  |
| **Sugars** | | | |
| H1 | Hexose | HMDB00122  HMDB00143  HMDB00169  HMDB00516  HMDB00660  HMDB01266  HMDB03345  HMDB03418  HMDB03449  HMDB12326  HMDB33704 | C00031  C00984  C00159  C00221  C02336  C08356  C00267  C00795  C00962  C15923  C01825 |
| **Amino acids** | | | |
| Arg | Arginine | HMDB00517  HMDB03416 | C00062  C00792 |
| Gln | Glutamine | HMDB00641  HMDB03423 | C00064  C00819 |
| Gly | Glycine | HMDB00123 | C00037 |
| His | Histidine | HMDB00177 | C00135 |
| Met | Methionine | HMDB00696 | C00073 |
| Orn | Ornithine | HMDB00214  HMDB03374 | C00515  C00077 |
| Phe | Phenylalanine | HMDB00159 | C00079 |
| Pro | Proline | HMDB00162  HMDB03411 | C00148  C00763 |
| Ser | Serine | HMDB00187  HMDB03406 | C00065  C00740 |
| Thr | Threonine | HMDB00167  HMDB04041 | C00188  C05519 |
| Trp | Tryptophan | HMDB00929  HMDB13609 | C00078  C00525 |
| Tyr | Tyrosine | HMDB00158 | C00082 |
| Val | Valine | HMDB00883 | C00183 |
| xLeu | xLeucine | HMDB00172 | C00407 |
| **Glycerophospholipids** | | | |
| lysoPC a C14:0 | lysoPhosphatidylcholine acyl C14:0 | HMDB10379 | C04230 |
| lysoPC a C16:0 | lysoPhosphatidylcholine acyl C16:0 | HMDB10382 | C04230 |
| lysoPC a C16:1 | lysoPhosphatidylcholine acyl C16:1 | HMDB10383 | C04230 |
| lysoPC a C17:0 | lysoPhosphatidylcholine acyl C17:0 | HMDB12108 | C04230 |
| lysoPC a C18:0 | lysoPhosphatidylcholine acyl C18:0 | HMDB10384  HMDB11128 | C04230 |
| lysoPC a C18:1 | lysoPhosphatidylcholine acyl C18:1 | HMDB02815  HMDB10385 | C04230 |
| lysoPC a C18:2 | lysoPhosphatidylcholine acyl C18:2 | HMDB10386 | C04230 |
| lysoPC a C20:3 | lysoPhosphatidylcholine acyl C20:3 | HMDB10393  HMDB10394 | C04230 |
| lysoPC a C20:4 | lysoPhosphatidylcholine acyl C20:4 | HMDB10395  HMDB10396 | C04230 |
| lysoPC a C24:0 | lysoPhosphatidylcholine acyl C24:0 | HMDB10405 | C04230 |
| lysoPC a C26:0 | lysoPhosphatidylcholine acyl C26:0 | HMDB29205 |  |
| lysoPC a C26:1 | lysoPhosphatidylcholine acyl C26:1 | HMDB29220 |  |
| lysoPC a C28:0 | lysoPhosphatidylcholine acyl C28:0 | HMDB29206 |  |
| lysoPC a C28:1 | lysoPhosphatidylcholine acyl C28:1 | HMDB29221 |  |
| lysoPC a C6:0 | lysoPhosphatidylcholine acyl C6:0 | HMDB29207 |  |
| PC aa C24:0 | Phosphatidylcholine diacyl C 24:0 |  |  |
| PC aa C26:0 | Phosphatidylcholine diacyl C 26:0 |  |  |
| PC aa C28:1 | Phosphatidylcholine diacyl C 28:1 |  |  |
| PC aa C30:0 | Phosphatidylcholine diacyl C 30:0 | HMDB07869  HMDB07934  HMDB07965 | C00157 |
| PC aa C30:2 | Phosphatidylcholine diacyl C 30:2 |  |  |
| PC aa C32:0 | Phosphatidylcholine diacyl C 32:0 | HMDB00564  HMDB07871  HMDB08031 | C00157 |
| PC aa C32:1 | Phosphatidylcholine diacyl C 32:1 | HMDB07872  HMDB07873  HMDB07969  HMDB08097 | C00157 |
| PC aa C32:2 | Phosphatidylcholine diacyl C 32:2 | HMDB07874  HMDB08002 | C00157 |
| PC aa C32:3 | Phosphatidylcholine diacyl C 32:3 | HMDB07876 | C00157 |
| PC aa C34:1 | Phosphatidylcholine diacyl C 34:1 | HMDB07971  HMDB07972  HMDB08003  HMDB08035  HMDB08100 | C00157 |
| PC aa C34:2 | Phosphatidylcholine diacyl C 34:2 | HMDB07973  HMDB08004  HMDB08005  HMDB08101  HMDB08133 | C00157 |
| PC aa C34:3 | Phosphatidylcholine diacyl C 34:3 | HMDB07974  HMDB07975  HMDB08006 | C00157 |
| PC aa C34:4 | Phosphatidylcholine diacyl C 34:4 | HMDB07883  HMDB07976 | C00157 |
| PC aa C36:0 | Phosphatidylcholine diacyl C 36:0 | HMDB07886  HMDB07977  HMDB08036  HMDB08265  HMDB08525 | C00157 |
| PC aa C36:1 | Phosphatidylcholine diacyl C 36:1 | HMDB08037  HMDB08038  HMDB08069  HMDB08102 | C00157 |
| PC aa C36:2 | Phosphatidylcholine diacyl C 36:2 | HMDB08039  HMDB08070  HMDB08135  HMDB00593 | C00157 |
| PC aa C36:3 | Phosphatidylcholine diacyl C 36:3 | HMDB07980  HMDB07981  HMDB08040  HMDB08105 | C00157 |
| PC aa C36:4 | Phosphatidylcholine diacyl C 36:4 | HMDB07982  HMDB08042  HMDB08106  HMDB08107  HMDB08138  HMDB08170  HMDB08203  HMDB08429 | C00157 |
| PC aa C36:5 | Phosphatidylcholine diacyl C 36:5 | HMDB07984  HMDB08015 | C00157 |
| PC aa C36:6 | Phosphatidylcholine diacyl C 36:6 | HMDB07892  HMDB08206 | C00157 |
| PC aa C38:0 | Phosphatidylcholine diacyl C 38:0 | HMDB07893  HMDB07985  HMDB08043  HMDB08267  HMDB08528  HMDB08755 | C00157 |
| PC aa C38:1 | Phosphatidylcholine diacyl C 38:1 | HMDB07894  HMDB07986  HMDB08044  HMDB08109  HMDB08268  HMDB08269 | C00157 |
| PC aa C38:3 | Phosphatidylcholine diacyl C 38:3 | HMDB08046  HMDB08047 | C00157 |
| PC aa C38:4 | Phosphatidylcholine diacyl C 38:4 | HMDB07988  HMDB08048  HMDB08112 | C00157 |
| PC aa C38:5 | Phosphatidylcholine diacyl C 38:5 | HMDB07989  HMDB07990  HMDB08050  HMDB08114 | C00157 |
| PC aa C38:6 | Phosphatidylcholine diacyl C 38:6 | HMDB07991  HMDB08083  HMDB08116  HMDB08147  HMDB08434  HMDB08499  HMDB08725 | C00157 |
| PC aa C40:1 | Phosphatidylcholine diacyl C 40:1 | HMDB07993  HMDB08052  HMDB08084  HMDB08117  HMDB08275 | C00157 |
| PC aa C40:2 | Phosphatidylcholine diacyl C 40:2 | HMDB08276  HMDB08308 | C00157 |
| PC aa C40:3 | Phosphatidylcholine diacyl C 40:3 |  |  |
| PC aa C40:4 | Phosphatidylcholine diacyl C 40:4 | HMDB08054  HMDB08279  HMDB08628 | C00157 |
| PC aa C40:5 | Phosphatidylcholine diacyl C 40:5 | HMDB08055  HMDB08056  HMDB08120 | C00157 |
| PC aa C40:6 | Phosphatidylcholine diacyl C 40:6 | HMDB08057  HMDB08089  HMDB08122 | C00157 |
| PC aa C42:0 | Phosphatidylcholine diacyl C 42:0 | HMDB08058  HMDB08282  HMDB08537  HMDB08760 | C00157 |
| PC aa C42:1 | Phosphatidylcholine diacyl C 42:1 | HMDB08059  HMDB08124  HMDB08283  HMDB08538  HMDB08762 | C00157 |
| PC aa C42:2 | Phosphatidylcholine diacyl C 42:2 | HMDB08570 |  |
| PC aa C42:4 | Phosphatidylcholine diacyl C 42:4 | HMDB08572 |  |
| PC aa C42:5 | Phosphatidylcholine diacyl C 42:5 | HMDB08287 | C00157 |
| PC aa C42:6 | Phosphatidylcholine diacyl C 42:6 | HMDB08288 | C00157 |
| PC ae C30:0 | Phosphatidylcholine acyl-alkyl C 30:0 | HMDB13341 |  |
| PC ae C30:1 | Phosphatidylcholine acyl-alkyl C 30:1 | HMDB13402 |  |
| PC ae C30:2 | Phosphatidylcholine acyl-alkyl C 30:2 |  |  |
| PC ae C32:1 | Phosphatidylcholine acyl-alkyl C 32:1 |  |  |
| PC ae C32:2 | Phosphatidylcholine acyl-alkyl C 32:2 |  |  |
| PC ae C34:0 | Phosphatidylcholine acyl-alkyl C 34:0 | HMDB13405 |  |
| PC ae C34:1 | Phosphatidylcholine acyl-alkyl C 34:1 |  |  |
| PC ae C34:2 | Phosphatidylcholine acyl-alkyl C 34:2 | HMDB11151 |  |
| PC ae C34:3 | Phosphatidylcholine acyl-alkyl C 34:3 | HMDB11211 |  |
| PC ae C36:0 | Phosphatidylcholine acyl-alkyl C 36:0 | HMDB13406  HMDB13417 |  |
| PC ae C36:1 | Phosphatidylcholine acyl-alkyl C 36:1 | HMDB13427 |  |
| PC ae C36:2 | Phosphatidylcholine acyl-alkyl C 36:2 | HMDB11243 |  |
| PC ae C36:3 | Phosphatidylcholine acyl-alkyl C 36:3 | HMDB13429 |  |
| PC ae C36:4 | Phosphatidylcholine acyl-alkyl C 36:4 | HMDB13435 |  |
| PC ae C36:5 | Phosphatidylcholine acyl-alkyl C 36:5 | HMDB11220 |  |
| PC ae C38:0 | Phosphatidylcholine acyl-alkyl C 38:0 | HMDB13408  HMDB13419 |  |
| PC ae C38:1 | Phosphatidylcholine acyl-alkyl C 38:1 | HMDB13430 |  |
| PC ae C38:2 | Phosphatidylcholine acyl-alkyl C 38:2 | HMDB13431 |  |
| PC ae C38:3 | Phosphatidylcholine acyl-alkyl C 38:3 | HMDB13439 |  |
| PC ae C38:4 | Phosphatidylcholine acyl-alkyl C 38:4 | HMDB13420 |  |
| PC ae C38:5 | Phosphatidylcholine acyl-alkyl C 38:5 | HMDB13432 |  |
| PC ae C38:6 | Phosphatidylcholine acyl-alkyl C 38:6 | HMDB13409 |  |
| PC ae C40:0 | Phosphatidylcholine acyl-alkyl C 40:0 | HMDB13421 |  |
| PC ae C40:1 | Phosphatidylcholine acyl-alkyl C 40:1 | HMDB13433 |  |
| PC ae C40:2 | Phosphatidylcholine acyl-alkyl C 40:2 | HMDB13437 |  |
| PC ae C40:3 | Phosphatidylcholine acyl-alkyl C 40:3 | HMDB13445 |  |
| PC ae C40:4 | Phosphatidylcholine acyl-alkyl C 40:4 | HMDB13442 |  |
| PC ae C40:5 | Phosphatidylcholine acyl-alkyl C 40:5 | HMDB13444 |  |
| PC ae C40:6 | Phosphatidylcholine acyl-alkyl C 40:6 | HMDB13422 |  |
| PC ae C42:0 | Phosphatidylcholine acyl-alkyl C 42:0 | HMDB13443 |  |
| PC ae C42:1 | Phosphatidylcholine acyl-alkyl C 42:1 | HMDB13434 |  |
| PC ae C42:2 | Phosphatidylcholine acyl-alkyl C 42:2 | HMDB13438 |  |
| PC ae C42:3 | Phosphatidylcholine acyl-alkyl C 42:3 | HMDB13459 |  |
| PC ae C42:4 | Phosphatidylcholine acyl-alkyl C 42:4 | HMDB13448 |  |
| PC ae C42:5 | Phosphatidylcholine acyl-alkyl C 42:5 | HMDB13451 |  |
| PC ae C44:3 | Phosphatidylcholine acyl-alkyl C 44:3 | HMDB13449 |  |
| PC ae C44:4 | Phosphatidylcholine acyl-alkyl C 44:4 | HMDB13453 |  |
| PC ae C44:5 | Phosphatidylcholine acyl-alkyl C 44:5 | HMDB13456 |  |
| PC ae C44:6 | Phosphatidylcholine acyl-alkyl C 44:6 | HMDB13457 |  |
| **Sphinglipids** | | | |
| SM (OH) C14:1 | Hydroxysphingomyeline C 14:1 |  |  |
| SM (OH) C16:0 | Hydroxysphingomyeline C 16:0 |  |  |
| SM (OH) C22:1 | Hydroxysphingomyeline C 22:1 |  |  |
| SM (OH) C22:2 | Hydroxysphingomyeline C 22:2 |  |  |
| SM (OH) C24:1 | Hydroxysphingomyeline C 24:1 |  |  |
| SM C16:0 | Sphingomyeline C 16:0 | HMDB10169 |  |
| SM C16:1 | Sphingomyeline C 16:1 | HMDB29216 |  |
| SM C18:0 | Sphingomyeline C 18:0 | HMDB01348 |  |
| SM C18:1 | Sphingomyeline C 18:1 | HMDB12100  HMDB12101 | C00550 |
| SM C20:2 | Sphingomyeline C 20:2 |  |  |
| SM C22:3 | Sphingomyeline C 22:3 |  |  |
| SM C24:0 | Sphingomyeline C 24:0 |  |  |
| SM C24:1 | Sphingomyeline C 24:1 | HMDB12107 | C00550 |
| SM C26:0 | Sphingomyeline C 26:0 | HMDB11698 |  |
| SM C26:1 | Sphingomyeline C 26:1 | HMDB13461 | C00550 |
